# Extracorporeal photochemotherapy induces bona fide immunogenic cell death

**DOI:** 10.1038/s41419-019-1819-3

**Published:** 2019-08-02

**Authors:** Kazuki Tatsuno, Takahiro Yamazaki, Douglas Hanlon, Patrick Han, Eve Robinson, Olga Sobolev, Alp Yurter, Felix Rivera-Molina, Najla Arshad, Richard L. Edelson, Lorenzo Galluzzi

**Affiliations:** 1 Department of Dermatology, Yale School of Medicine, New Haven, CT USA; 2 Department of Radiation Oncology, Weill Cornell Medical College, New York, NY USA; 3 Department of Cell Biology, Yale School of Medicine, New Haven, CT USA; 4 Department of Immunobiology, Yale School of Medicine, New Haven, CT USA; 5 Comprehensive Cancer Center, Yale University School of Medicine, New Haven, CT USA; 6 Sandra and Edward Meyer Cancer Center, New York, NY USA; 7 Université Paris Descartes/Paris V, Paris, France

**Keywords:** Tumour immunology, Cell death, Immune cell death

## Abstract

Extracorporeal photochemotherapy (ECP) is employed for the management of cutaneous T cell lymphoma (CTCL). ECP involves the extracorporeal exposure of white blood cells (WBCs) to a photosensitizer, 8-methoxypsoralen (8-MOP), in the context of ultraviolet A (UVA) radiation, followed by WBC reinfusion. Historically, the therapeutic activity of ECP has been attributed to selective cytotoxicity on circulating CTCL cells. However, only a fraction of WBCs is exposed to ECP, and 8-MOP is inactive in the absence of UVA light, implying that other mechanisms underlie the anticancer effects of ECP. Recently, ECP has been shown to enable the physiological differentiation of monocytes into dendritic cells (DCs) that efficiently cross-present tumor-associated antigens (TAAs) to CD8^+^ T lymphocytes to initiate cognate immunity. However, the source of TAAs and immunostimulatory signals for such DCs remains to be elucidated. Here, we demonstrate that 8-MOP plus UVA light reduces melanoma cell viability along with the emission of ICD-associated danger signals including calreticulin (CALR) exposure on the cell surface and secretion of ATP, high mobility group box 1 (HMGB1) and type I interferon (IFN). Consistently, melanoma cells succumbing to 8-MOP plus UVA irradiation are efficiently engulfed by monocytes, ultimately leading to cross-priming of CD8^+^ T cells against cancer. Moreover, malignant cells killed by 8-MOP plus UVA irradiation in vitro vaccinate syngeneic immunocompetent mice against living cancer cells of the same type, and such a protection is lost when cancer cells are depleted of calreticulin or HMGB1, as well as in the presence of an ATP-degrading enzyme or antibodies blocking type I IFN receptors. ECP induces bona fide ICD, hence simultaneously providing monocytes with abundant amounts of TAAs and immunostimulatory signals that are sufficient to initiate cognate anticancer immunity.

## Background

The term extracorporeal photochemotherapy (ECP) refers to a therapeutic procedure in which cutaneous T cell lymphoma (CTCL) patients are subjected to leukapheresis followed by: (1) extracorporeal exposure or white blood cells (WBCs) to 8-methoxypsoralen (8-MOP) in the context of ultraviolet A (UVA) irradiation, and (2) WBC reinfusion^1^. Only in the presence of UVA light, 8-MOP transiently acquires an activated chemical configuration that enables the formation of DNA monoadducts or interstand crosslinks^2^. These photolesions are recognized and potentially repaired by the nucleotide excision repair (NER) pathway, unless they drive replication fork collapse, a situation that generally engages double-strand break (DSB) repair^3^. That said, 8-MOP can be finely titrated to cause sufficient amounts of photoadducts to overwhelm the DNA repair machinery, ultimately leading to a rather slow wave of regulated cell death (RCD) that develops over the 3–4 days after ECP^2,4,5^. Importantly, not all cell types exhibit comparable sensitivity to 8-MOP plus UVA light^6^. In particular, circulating lymphocytes appear to be considerably more sensitive than monocytes to RCD driven by 8-MOP plus UVA irradiation^7^.

Such a differential sensitivity has frequently been invoked to explain the therapeutic activity of ECP against CTCL patients^8^. However, only a relatively small fraction (<20%) of circulating WBCs are actually exposed to 8-MOP plus UVA light in the course of ECP^1,9^, and the disappearance of untreated malignant cells suggest the elicitation of antigen-specific immunity^10^. Moreover, it has recently been reported that ECP enables the physiological differentiation of monocytes into dendritic cells (DCs) as a consequence of monocyte-platelet interactions^11,12^, and that such DCs are highly efficient at cross-presenting cancer-associated antigens to CD8^+^ T lymphocytes to initiate cognate anticancer immunity^13^. However, the mechanism whereby ECP provides DCs with sufficient amounts of antigenic material from cancer cells in the context of immunostimulatory signals remains to be determined.

We, therefore, tested the possibility that ECP would drive a particularly immunogenic variant of apoptotic cell death that is commonly known as immunogenic cell death (ICD)^5,14^. The high immunostimulatory potential of ICD depends on the spatiotemporally defined emission of a variety of damage-associated molecular patterns (DAMPs), which for the most part operate as pro-phagocytic, chemotactic and/or activatory signals for DCs or their precursors^14^. These DAMPs include (but are not limited to): (1) calreticulin (CALR), an endoplasmic reticulum (ER) chaperone that—upon exposure on the plasma membrane of dying cells—favors their phagocytosis^15,16;^ (2) ATP, which is secreted by cells undergoing ICD in an autophagy-dependent manner, and ultimately acts as chemoattractant—via purinergic receptor P2Y2 (P2RY2)—or activation signal—via purinergic receptor P2X 7 (P2RX7)^17,18;^ (3) high mobility group box 1 (HMGB1), a nuclear protein that—upon release by cells succumbing to ICD—mediates adjuvant-like effects by binding to Toll-like receptor 4 (TLR4)^19;^ and (4) type I interferon (IFN), a cytokine that is secreted in the context of ICD and ultimately mediates chemotactic and immunostimulatory effects via interferon alpha and beta receptor (IFNARs) expressed on both cancer and immune cells^20,21^. Accumulating preclinical and clinical evidence indicates that ICD as well as ICD-associated DAMP emission and detection impact disease outcome in a variety of tumors^22^, rendering this functionally unique RCD modality a particularly promising target for therapeutic purposes.

Here, we report for the first time that ECP causes loss of cancer cell viability in the context of ICD-associated DAMP release, resulting in the uptake of dying cancer cells by monocytes and robust cross-priming of tumor-specific CD8^+^ T cells. Consistently, mouse cancer cells succumbing to 8-MOP plus UVA light in vitro were sufficient to vaccinate tumor-naive, syngeneic, immunocompetent mice against a subsequent challenge with living cancer cells of the same type, an effect that was lost when ICD-associated DAMP emission was blocked by pharmacological or genetic interventions. In summary, this is the first demonstration that ECP drives bona fide ICD, which may explain the robust therapeutic activity of ECP in CTCL patients.

## Materials and methods

### Cell culture

Culture media and supplements were obtained from Invitrogen, unless otherwise noted. Mouse melanoma B16 cells (kindly provided by Dr. Bosenberg, Yale University) were grown in DMEM supplemented with 10% heat-inactivated fetal bovine serum (FBS) and 1% penicillin/streptomycin. B16 cells expressing truncated chicken ovalbumin (B16-OVA clone M05.F5, kindly provided by Dr. Falo Jr., University of Pittsburgh School of Medicine) were maintained in RPMI supplemented with 10% FBS, 10 mM HEPES, 1% non-essential amino-acids, 2 mM l-glutamine, 1 mM sodium pyruvate, 0.05 mM β-mercaptoethanol, 1% penicillin/streptomycin, and 1 mg/mL G418. Human melanoma YUCOT cells (kindly provided by the Core of the Yale SPORE in Skin Cancer) were grown in OptiMEM media supplemented with 5% FBS and 1% penicillin/streptomycin. Mouse colorectal carcinoma MC38 cells (kindly provided by Dr. Hynes from, Massachusetts Institute of Technology, with permission of Dr. Varki, University of California, San Diego) were grown in DMEM supplemented with 10% FBS, 10 mM HEPES, 1% non-essential amino-acids, 2 mM l-glutamine, 1 mM sodium pyruvate, and 1% penicillin/streptomycin. All cell lines were cultured at 37 °C, 5% CO_2_ and routinely tested for mycoplasma contamination by standard PCR methods.

### Generation of CALR- and HMGB1-depleted cells

Puromycin selection-based pLKO.1 lentiviral vectors expressing a short-hairpin RNA (shRNA) specific for mouse CALR (target: CCCTGCCATCTATTTCAAAGA) or HMGB1 (target: GAAGATGATGATGATGAATA) were utilized to generate CALR- or HMGB1-depleted B16-OVA cells. shRNAs were provided by the Functional Genomics Shared Resource Core of the Yale Cancer Center. The lentiviral vector SHC001 expressing a non-targeting shRNA (MISSION, from Sigma, Cat# SHC016) was utilized as a negative control. Vectors were packaged in ViraPower™ Lentiviral Packaging Mix kit (ThermoFisher, Cat # K497500), or in the psPAX2 and pMD2G plasmids, followed by infection of human embryonic kidney HEK293T cells for viral propagation. Culture medium from infected cells was collected and filtered with Millex-GV 33 mm PVDF filter (Millipore, Cat# SLGV033RS) and then concentrated with Amicon Ultra-15 centrifugal filters (Millipore, Cat# UFC910024). Finally, B16-OVA cells were infected with lentiviruses, selected with puromycin for 2 days, and then processed for knockdown confirmation according to standard immunoblotting procedures^23^, with antibody specific for CALR (Abcam, Cat# AB2907), HGMB1 (Abcam, Cat# AB227168) and actin beta 1 (ACTB1, from Sigma, Cat# A5316) or heat shock protein 90, beta (Grp94), member 1 (HSP90B1, from Enzo Life Sciences, Cat# ADI-SPA-850) as loading controls.

### Cell death induction and assessment

Cancer cells were either (1) subjected to 3 cycles of freeze/thawing in dry-ice cooled methanol and 37 °C water bath, (2) exposed to cis-diammineplatinum dichloride (CDDP, from Sigma, Cat# P4394) in culture media, or (3) optionally pre-incubated with 200 ng/mL 8-methoxypsoralen (8-MOP; from UVADEX; Therakos) for 20 min and then subjected to UVA irradiation. Cell number was assessed with the CellTiter Blue Kit (Promega, Cat# G8080), as per manufacturer instructions. Apoptotic cell death was measured by standard protocols^24^, based on the FITC-Annexin V Apoptosis Detection Kit (Biolegend, Cat# 640914), as per the manufacturer. Clonogenic assays were performed by plating cancer cells in 6-well plates (50 cells/well), followed by 7 days of standard culture, fixation/staining in 1% crystal violet (Fisher Science, Cat# S25275A), and manual quantification.

### Detection of surface-exposed CALR

B16-OVA cells were harvested and washed with ice-cold PBS then incubated with rabbit anti-CALR antibody (Abcam, Cat# AB2907) at 1:100 dilution in FACS buffer at 4 °C for 1 h. Next, cells were stained with anti-rabbit IgG Alexa Fluor488 conjugates (Invitrogen, Cat# A11070) at 1:750 dilution for 30 min, followed by addition of propidium iodide (PI, from Sigma, Cat# P4864) at 0.5 mg/mL final concentration and acquisition by flow cytometry. As per gold-standard recommendations^25^, PI^+^ cells were excluded from the analysis.

### Detection of soluble DAMPs

Supernatants from B16-OVA cells maintained in control conditions or exposed to cytotoxic stimuli as described above were collected, cleared of cellular fragments by centrifugation and snap-frozen. HMGB1 was quantified with the HMGB1 ELISA Kit (Tecan, Cat# ST51011), ATP with the luciferase-based Enliten ATP Assay (Promega, Cat# FF2000) and type I IFN with the Mouse IFN Beta ELISA Kit (PBL, Cat# 42410-1), all according to manufacturer’s instructions.

### Phagocytosis assays

Peripheral blood was collected from healthy volunteers into 1:100 5000 U/mL heparin (McKesson Packaging Services, Cat# 949512), and platelet-containing peripheral blood mononuclear cells (PBMCs) were isolated by density gradient centrifugation over Isolymph (CTL Scientific Supply Corp., Cat# 50-300-403) following manufacturer’s instructions. PBMCs were then co-cultured for 24 h with YUCOT cells pre-labeled with PKH green (Sigma, Cat# PKH67GL) and exposed to cytotoxic stimuli as described above (at 2:1 ratio, in culture medium containing autologous plasma). Lysotracker Red (ThermoFisher, Cat# L7528) was added 10 h before the end of the experiment. At endpoint, cells were stained with Alexa Fluor647-labeled anti-human CD14 antibody (Biolegend, Cat# 325611) and plated onto Ibidi μ-slides VI (Ibidi, Cat# 80606) prior to imaging. Cells were imaged on a Yokogawa-type Spinning-Disk Confocal Microscope (PerkinElmer) mounted on an inverted microscope base (IX-71, Olympus) equipped with a 1 Kb × 1 Kb electron-multiplying CCD camera (Hamamatsu Photonics) and a temperature-controlled stage set, under control by the Ultraview ERS software (PerkinElmer). Cells were imaged on a ×60 1.4 NA oil objective lens with a pixel size of 0.14 µm using 5 solid-state lasers: 405-, 488-, 561-, 594- and 640-nm (Melles Griot). Image processing and data analysis to calculate and evaluate the number of GFP^+^ and RFP^+^ positive fragments within CD14^+^ cells were conducted using Volocity Software.

### Animals

C57BL/6J, B6.SJL-Ptprca Pepcb/BoyJ (B6.SJL CD45.1) mice and C57BL/6-Tg(TcraTcrb) mice, which recognize the OVA peptide in the context of H2Kb (OT-I CD45.2), were purchased from Jackson Laboratory. Age and sex-matched mice that were at least 6 weeks of age were used.

### Isolation of murine PBMCs

Peripheral blood was collected from mice via non-lethal bleeding of the superficial temporal vein or retro-orbital plexis. Whole blood was collected into 1:100 5000 U/mL heparin (McKesson Packaging Services), and platelet-containing PBMCs were isolated via Lympholyte M gradient separation (Cedarlane Labs, Cat# CL5035) followed by treatment with ACK buffer (Lonza, Cat# 10-548E) to eliminate residual red blood cells.

### OT-I proliferation assays

On day 1, PBMCs were cultured at 2.5 × 10^6^ cells/mL overnight in phenol red-free RPMI supplemented with 15% autologous mouse serum, 1% l-glutamine and 1% penicillin/streptomycin, in the optional presence of 50 μg/mL recombinant OVA or 1.25 × 10^6^ B16 or B16-OVA cells treated with cytotoxic stimuli as described above. On day 2, cells were harvested, washed in PBS, and re-plated in 96-well plates (2 × 10^5^ cells/well). Subsequently, 1 × 10^5^ OT-I CD8^+^ T cells pre-labeled with 5(6)-carboxyfluorescein diacetate *N*-succinimidyl ester (CFSE, from Thermo-Fischer, Cat# C34554) were added to each well. CFSE labeling was performed as follows: splenic extracts were subjected to osmotic shock with ACK lysis buffer (Lonza) to remove red blood cells, followed by splenocyte resuspension in medium supplemented with 10 μM CFSE for 15 min at 37 °C. The reaction was quenched using 5× volume of RPMI supplemented with 10% FBS, cells were pelleted, and washed again with PBS to remove free dyes. CD8^+^ cells were then isolated using magnetic nanoparticle negative selection kits (EasySep, from Stem Cell, Cat# 19853), according to the manufacturer’s instructions. On day 4, cells were harvested and analyzed by flow cytometry for CFSE dilution.

### Vaccination assays

One × 10^6^ tumor cells subjected to cytotoxic stimuli as described above were resuspended in 100 μL PBS and inoculated s.c. into the lower flank of 7-week-old female C57BL/6J mice (vaccination). The same volume of PBS was used as a negative control. One week later, mice received 2 × 10^5^ untreated cancer cells of the same type, s.c., into the contralateral flank (challenge). Tumor incidence and growth was monitored routinely with a common caliper up to 60-days post-challenge. Mice were euthanized during the observation period when tumor size exceed ethical limits or mice exhibited signs of systemic disease (hunched posture, anorexia, weight loss). Mice rejecting the challenge injection were optionally re-challenged 60-days later with homologous or heterologous cancer cells. Tumor-free survival (TFS) is reported.

## Results

### ECP mediates cytostatic and cytotoxic effects against melanoma cells

**Fig. 1:**
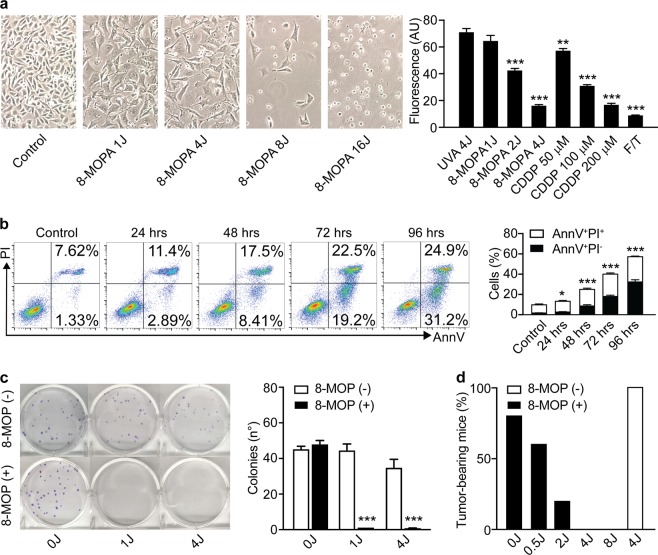
8-MOP plus UVA light mediates dose- and time-dependent cytotoxic activity against melanoma cells in vitro. **a** Residual number of B16-OVA cells upon exposure to 8-methoxypsoralen (8-MOP) in the context of UVA light at the indicated dose, 4J UVA irradiation only, or the indicated dose of cisplatin (CDDP) for 48 h, or upon exposure to repeated freeze/thawing (F/T) and culture in control conditions for 48 h. Representative micrographs and quantitative data are reported. *n* = 3; **p < 0.01, ***p < 0.001 (Student’s *t*-test), as compared to cells receiving 4J UVA light only. **b** Percentage of AnnV^+^PI^−^ and AnnV^+^PI^+^ B16-OVA cells upon exposure to 8-MOP plus 4J UVA light (8-MOPA) and culture in control conditions for the indicated time. Representative dotplots with percentages of events in relevant quadrants, and quantitative results are reported. *n* = 3; *p < 0.05, ***p < 0.001 (Student’s *t*-test), as compared to untreated cells. **c** Residual clonogenic potential of B16-OVA cells optionally exposed to 8-MOP in the context of no irradiation or UVA light at the indicated dose. Representative images and quantitative results are reported. *n* = 3; ***p < 0.001 (Student’s *t*-test), as compared to cells exposed to the same UVA dose in the absence of 8-MOP. **d** Percentage of C57BL/6 mice developing tumors upon subcutaneous inoculation of B16-OVA cells exposed in vitro to the indicated UVA dose in the presence or absence of 8-MOP. Quantitative results from one representative experiment involving 10 mice per group are reported

To investigate the ability of 8-MOP plus UVA irradiation to mediate cytotoxic effects in vitro, we harnessed four different experimental systems. First, we employed a fluorometric assay for cell number on ovalbumin (OVA)-expressing mouse melanoma B16 (B16-OVA) cells exposed to 8-MOP plus UVA light at different therapeutically relevant doses (1J – 4J) for 48 h, as compared to (1) B16 cells receiving 4J UVA light in the absence of 8-MOP; (2) B16 cells responding to the chemotherapeutic agent cisplatin (CDDP), which is known to mediate a non-immunogenic variant of RCD;^26^ and (3) B16 cells subjected to freeze–thawing cycles, which cause a rapid necrotic response with tolerogenic, rather than immunogenic, consequences^27,28^. As expected, 4J UVA light-mediated little effects in the absence of 8-MOP. Conversely, 8-MOP plus 4J UVA irradiation caused a cytostatic response as pronounced as that caused by a supratherapeutic dose of CDDP (200 µM) (Fig. 1a). Conversely, 8-MOP plus 1J or 2J UVA light exerted minimal or intermediate cytostatic effects, in thus far resembling 50 µM and 100 µM CDDP, respectively (Fig. 1a).

We next set to interrogate biochemical biomarkers of apoptotic cell death in B16-OVA cells exposed to 8-MOP plus 4J UVA light in vitro over time. In particular, we monitored the apoptosis-associated exposure of phosphatidylserine (PS) on the outer leaflet of the plasma membrane, which is indicative of apoptotic caspase activation, and the uptake of the vital die propidium iodide (PI), which is indicative of plasma membrane permeabilization^29^. In line with the previous reports^4^, we found that 8-MOP plus 4J UVA causes a slow apoptotic response that—by 96 h after treatment—involves ~60% of the cell population (Fig. 1b). The remaining 40% of B16-OVA cells exposed to 8-MOP plus 4J UVA, however, die with a delayed kinetic or are permanently unable to proliferate, as demonstrate by long-term clonogenic assays (Fig. 1c). Of note, 8-MOP plus 1J UVA light also compromise completely the ability of B16-OVA cells to form colonies (Fig. 1c), suggesting that despite a limited cytostatic response at 48 h, 8-MOP plus UVA doses <4J are also efficient at inducing delayed RCD or cellular senescence in vitro.

To further validate these findings, we tested the ability B16-OVA cells exposed to various UVA doses plus 8-MOP in vitro to generate tumors upon subcutaneous inoculation into syngeneic immunocompetent C57BL/6 mice. As expected, B16-OVA cells receiving 4J UVA light in the absence of 8-MOP exhibited intact tumorigenic potential (Fig. 1d). Conversely, the ability of B16-OVA cells to generate neoplastic lesions in syngeneic immunocompetent hosts was fully compromised upon exposure to 4J or 8J UVA light plus 8-MOP, but only partially so when 8-MOP was delivered in the context 0.5J or 2J UVA irradiation (Fig. 1d). Thus, when B16-OVA cells exposed to 8-MOP plus UVA doses <4J in vitro are allowed to respond to stress in vivo, some degree of clonogenic potential is rescued. Altogether, these data prompted us to select the UVA dose of 4J for subsequent studies.

### ECP-driven RCD is associated with emission of ICD-associated danger signals, improved phagocytosis of cell corpses and robust cross-priming of tumor-specific lymphocytes

**Fig. 2:**
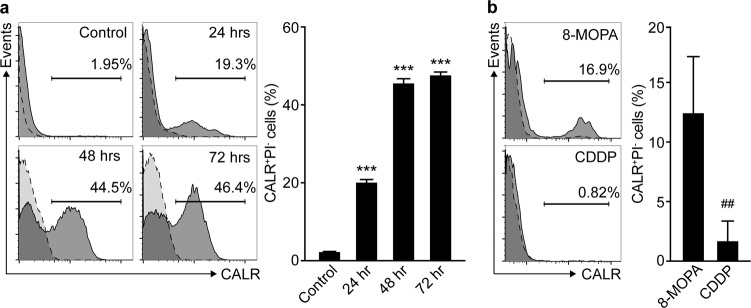
Melanoma cells treated with 8-MOP plus UVA irradiation expose CALR on the cells surface. **a** Percentage of CALR^+^PI^−^ B16-OVA cells upon exposure to 8-methoxypsoralen (8-MOP) plus 4J UVA light (8-MOPA) and cultured in control conditions for the indicated time. Representative histograms (isotype staining is reported as dashed profile) and quantitative results are reported. *n* = 3; ***p < 0.001 (Student’s *t*-test), as compared to untreated cells. **b** Percentage of CALR^+^PI^-^ B16-OVA cells upon exposure to 8-MOPA and cultured in control conditions for 24 h, or culture in the presence of 200 µM cisplatin (CDDP) for 24 h. Representative histograms (isotype staining is reported as dashed profile) and quantitative results are reported. *n* = 3; ^##^*p* < 0.01 (Student’s *t*-test), as compared to cells exposed to 8-MOPA

**Fig. 3:**
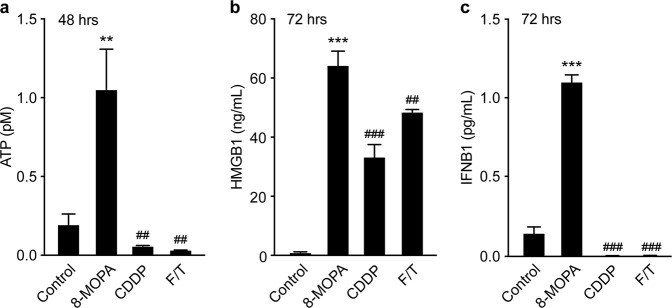
Melanoma cells responding to 8-MOP plus UVA light in vitro release soluble DAMPs. **a**–**c**. Amounts of ATP (**a**), high mobility group box 1 (HMGB1; **b**) and interferon beta 1 (IFNB1; **c**) in the supernatants of B16-OVA cells maintained in control conditions or treated with 200 µM cisplatin (CDDP) for the indicated time, or B16-OVA cells subjected to repeated freeze/thawing (F/T) or exposed to 8-methoxypsoralen plus 4J UVA light (8-MOPA) and then cultured in control conditions for the indicated time. *n* = 8; **p < 0.01, ***p < 0.001 (Student’s *t*-test), as compared to untreated cells; ^##^*p* < 0.01, ^###^*p* < 0.001 (Student’s *t*-test), as compared to cells exposed to 8-MOPA

Next, we set to investigate whether RCD driven by 8-MOP plus 4J UVA light is accompanied by the emission of the DAMPs that underlie the ability of cell death to be perceived as immunogenic, namely CALR exposure, ATP secretion, HMGB1 release and type I IFN secretion^14^. 8-MOP plus 4J UVA light induced the appearance of a population of B16-OVA cells staining positively for CALR but negatively for PI (indicating that the CALR-specific antibody is bound to the surface of intact cells)^25^ that progressively increased up to ~45% of PI^−^ cells over 72 h (Fig. 2a). At 24 h, the percentage of CALR^+^PI^−^ cells accumulating in response to 8-MOP plus 4J UVA irradiation compared favorably with that caused by CDDP treatment (Fig. 2b). Along similar lines, 8-MOP plus 4J UVA light stimulated ATP secretion (measured 48 h after treatment), as well as HMGB1 and type I IFN release (measured 72 h after treatment) by cultured B16-OVA cells (Fig. 3a–c). Conversely, B16-OVA cells responding to CDDP or subjected to freeze–thawing cycles in vitro not only failed to secrete ATP and type I IFN (two active biological processes), but also released limited amounts of HMGB1 (which in multiple instances occurs passively, as a simple consequence of cellular breakdown) (Fig. 3a–c). In summary, the demise of B16-OVA cells caused by 8-MOP plus 4J UVA light in vitro is accompanied by the emission of ICD-associated danger signals.

**Fig. 4:**
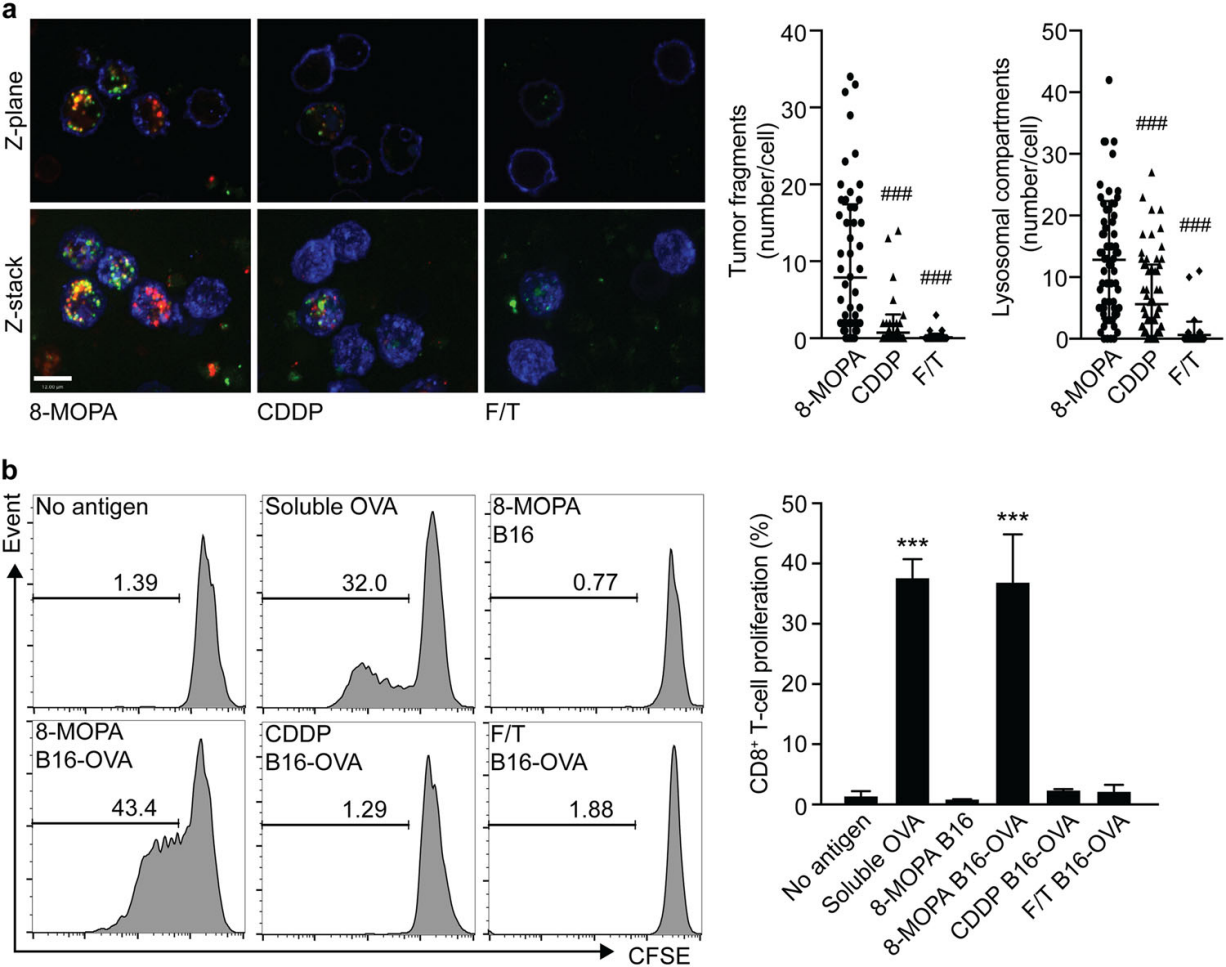
Monocytes exposed to malignant cells pre-treated with 8-MOP plus UVA irradiation exhibit robust phagocytic and cross-priming activity. **a** Number of PHK-labeled tumor fragments and lysosomes observed within human peripheral blood mononuclear cells (PBMCs) upon exposure to YUCOT cells previously subjected to repeated freeze/thawing (F/T) or exposed to 8-methoxypsoralen plus 4J UVA light (8-MOPA) and then cultured in control conditions for 24 h, or treated with 200 µM cisplatin (CDDP) for 24 h. Representative images and quantitative results are reported. *n* = 65 cells (8-MOPA), 82 cells (CDDP), 47 cells (F/T); ^###^*p* < 0.001 (Student’s *t*-test), as compared to cells exposed to 8-MOPA. Scale bar = 14 µm. **b** Percentage of proliferating OT-I cells upon overnight exposure to PBMCs in the indicated cross-priming conditions. Representative histograms and quantitative results are reported. *n* = 3; ***p < 0.001 (Student’s *t*-test), as compared to OT-I cells cultured with PBMCs and no antigen

We next set to explore the ability of ICD-associated DAMPs emitted by cancer cells succumbing to 8-MOP plus 4J UVA irradiation in vitro to drive immunobiological processes linked to the activation of cognate anticancer immunity. To this aim, we labeled human melanoma YUCOT cells with a PKH fluorescent dye (which stains the plasma membrane), treated them with 8-MOP plus 4J UVA light, CDDP, or repeated freeze–thawing, and then we co-cultured them with human donor-derived peripheral blood mononuclear cells (PBMCs). The cell mixture was further labeled with a dye for lysosomal/endosomal compartments and allowed to incubate overnight before the addition of a CD14-specific antibody (to identify PBMCs). Whereas the corpses of YUCOT cells treated with CDDP or subjected to freeze–thawing cycles were virtually not engulfed by monocytes, the debris of YUCOT cells responding to 8-MOP plus 4J UVA light were taken up efficiently, and often co-localized with expanded acidic cellular compartments compatible with lysosomes or late endosomes (Fig. 4a). These findings are consistent with previous observations demonstrating that prolonged interactions between dying cancer cells and newly formed DCs increase the potency of ECP^30^. Furthermore, B16-OVA cells treated with 8-MOP in the context of 4J UVA irradiation and co-cultured overnight with syngeneic PBMCs were efficiently cross-presented to OT-I T cells (which are genetically modified to express an OVA-specific TCR)^31^ pre-stained with 5(6)-carboxyfluorescein diacetate *N*-succinimidyl ester (CFSE), resulting in CFSE dilution (a marker of T cell proliferation) in up to 40% OT-I cells (Fig. 4b). Soluble OVA had similar potency in this assay (Fig. 4b). Conversely, untreated B16-OVA cells, B16-OVA cells subjected to repeated freeze–thawing and B16-OVA cells succumbing to CDDP administration were completely unable to initiate cross-priming and consequent OT-I cell proliferation (Fig. 4b). Taken together, these findings demonstrate that melanoma cells succumbing to 8-MOP plus 4J UVA light are efficiently engulfed by monocytes to drive the cross-priming of tumor-specific T lymphocytes.

### ECP drives bona fide ICD

**Fig. 5:**
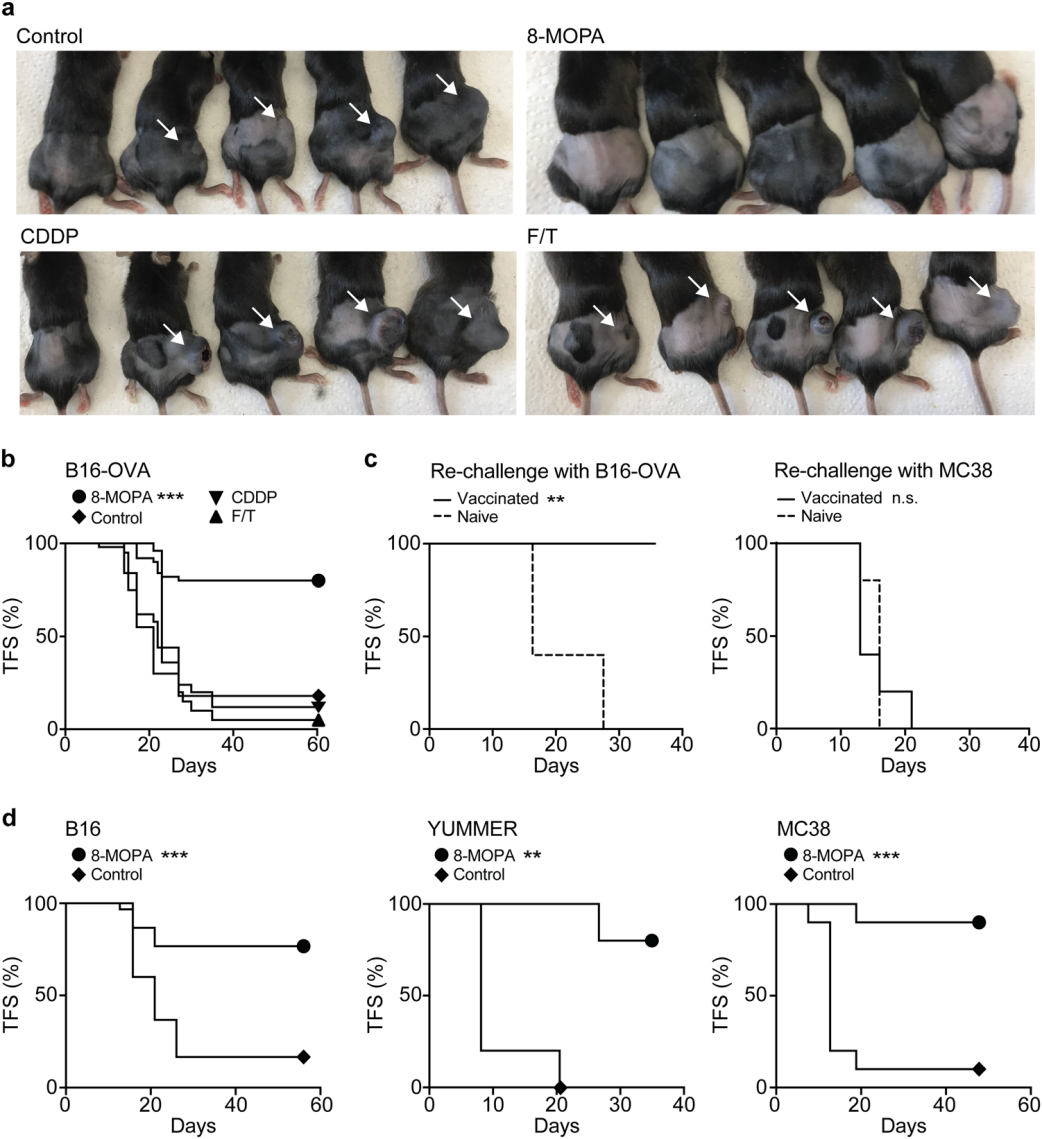
Malignant cells treated with 8-MOP plus UVA light in vitro confer tumor-specific immunological protection upon inoculation in syngeneic immunocompetent hosts. **a**, **b** Tumor-free survival (TFS) of C57BL/6 mice upon inoculation of PBS (no vaccination) or B16-OVA cells subjected to repeated freeze/thawing (F/T) or exposed to 8-methoxypsoralen plus 4J UVA light (8-MOPA) and then cultured in control conditions for 24 h, or treated with 200 µM cisplatin (CDDP) for 24 h, followed (one week later) by the contralateral inoculation of living B16-OVA cells. Representative images (**a**) and quantitative results (**b**) are reported. *n* = 50 mice (8-MOPA), 50 mice (control), 25 mice (CDDP), 20 mice (F/T); ***p < 0.001 (Log-rank test), as compared to mice vaccinated with PBS. **c** Percentage of tumor-naive C57BL/6 mice and C57BL/6 mice pre-vaccinated with B16-OVA cells exposed to 8-MOPA and cultured in control conditions for 24 h developing tumors upon re-challenge with B16-OVA cells or MC38 cells. Quantitative results are reported. *n* = 5 mice per group; **p < 0.01, n.s., not significant (Log-rank test), as compared to tumor-naive mice. **d** TFS of C57BL/6 mice upon inoculation of PBS (no vaccination) or wild-type B16, YUMMER, or MC38 cells exposed to 8-MOPA and cultured in control conditions for 24 h, followed (one week later) by the contralateral inoculation of living B16, YUMMER or MC38 cells, respectively. Quantitative results are reported. *n* = 30 mice per group (B16), 5 mice per group (YUMMER), 10 mice per group (MC38); **p < 0.01, ***p < 0.001 (Log-rank test), as compared to mice vaccinated with PBS

To determine whether cancer cells responding to 8-MOP plus 4J UVA irradiation in vitro are sufficient to initiate cognate anticancer immunity, we harnessed gold-standard vaccination assays^25^. In this setting, dying malignant cells are inoculated subcutaneously into syngeneic immunocompetent mice, in the absence of any drug or immunological adjuvant, as a prophylactic vaccination. One-to-weeks later, the same mice are challenged with living cancer cells of the same type in the contralateral flank, and tumor incidence is monitored over time. As a control for the intact tumorigenic potential of the challenge injection, unvaccinated mice are employed. As per previous reports^15,32^, neither freeze-thawed B16-OVA cells nor B16-OVA cells succumbing to CDDP in vitro were able to generate protective immunity once inoculated into immunocompetent C57BL/6 mice (Fig. 5a, b). Conversely, B16-OVA cells responding to 8-MOP plus 4J UVA irradiation in vitro conferred immunological protection against the establishment of novel B16-OVA lesions to up to 80% of the mice (Fig. 5a, b). Importantly, the protection afforded by B16-OVA cells succumbing to 8-MOP plus 4J light was tumor-specific, as vaccinated mice could not reject a challenge with MC38 cells, in thus far resembling tumor-naive animals (Fig. 5c). Comparable vaccination efficacy was obtained with wild-type B16 cells, with mouse melanoma YUMMER cells, as well as with mouse colorectal carcinoma MC38 cells (Fig. 5d). These data demonstrate that ECP drives bona fide ICD.

Interestingly, we noted that 8-MOP combined with 16J UVA light exerts a superior cytotoxic effect as compared to 8-MOP plus 4J UVA irradiation, but this is linked to decreased (rather than increased) DAMP emission, with the exception of HMGB1 release (which is known to correlate with cytotoxicity) (Supplementary Fig. 1A, B). Consistently, B16-OVA cells responding to 8-MOP plus 16J UVA light in vitro are less efficient at vaccinating C57BL/6 as compared to the same cells exposed to 8-MOP plus 4J UVA irradiation (Supplementary Fig. 1C). These data are in line with the notion that ICD has a precise timing that cannot be substituted for by a mixed of cellular components such as those obtained by repeated freeze–thawing or 8-MOP in the presence of high UVA doses.

**Fig. 6:**
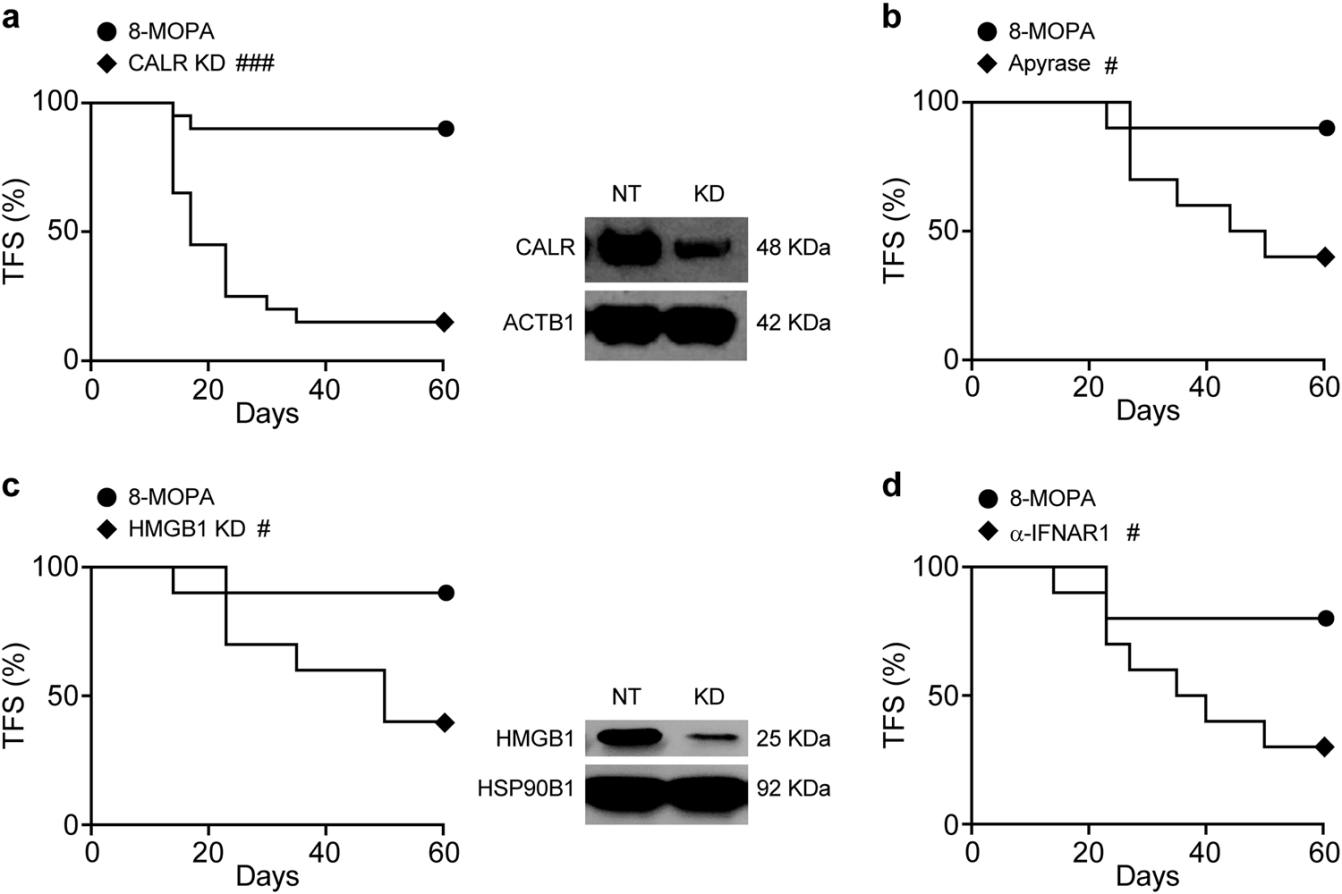
Immunological protection by malignant cells treated with 8-MOP plus UVA irradiation in vitro depends on ICD-associated DAMP emission. **a**–**d** Tumor-free survival (TFS) of C57BL/6 mice upon vaccination with B16-OVA cells expressing a non-targeting (NT) shRNA (**a**, **c**) or wild-type B16-OVA cells (**b**, **d**) exposed to 8-methoxypsoralen plus 4J UVA light (8-MOPA) and cultured for 24 h in control conditions (**a**–**d**) or in the context of calreticulin (CALR) shRNA-mediated knockdown (KD) (**a**), apyrase treatment (**b**), high mobility group box 1 (HMGB1) shRNA-mediated KD (**c**), or interferon alpha and beta receptor subunit 1 (IFNAR1) blockage (**d**), followed (one week later) by the contralateral inoculation of living B16-OVA cells. Representative images and quantitative results are reported. *n* = 20 mice per group (CALR knockdown), 10 mice per group (apyrase treatment), 10 mice per group (HMGB1 knockdown), 10 mice per group (IFNAR1 blockage); ^#^*p* < 0.05, ^###^*p* < 0.001 (Log-rank test), as compared to mice vaccinated with B16-OVA cells exposed to 8-MOPA in control conditions. Insets in **a** and **c** depict efficacy of CALR and HMGB1 depletion, respectively. Actin beta 1 (ACTB1) and heat shock protein 90, beta (Grp94), member 1 (HSP90B1) were monitored as loading controls

To obtain robust mechanistic insights into ECP-driven ICD and compare it with chemotherapy-, radiotherapy- and photodynamic therapy-driven ICD, each of which is associated with precise molecular signatures^14^, we employed pharmacological and genetic interventions that block DAMP emission by malignant cells exposed to 8-MOP plus 4J UVA irradiation in vitro and tested residual immunogenic potential in vivo, in gold-standard vaccination assays. We found that the immunological protection afforded to C57BL/6 mice by B16-OVA cells succumbing to 8-MOP plus 4J UVA light is largely compromised not only by the short-hairpin RNA (shRNA)-mediated depletion of CALR or HMGB1 in cancer cells, but also by the pharmacological degradation of extracellular ATP with apyrase and the blockage of IFNAR1 with a specific monoclonal antibody (Fig. 6a–d). These findings demonstrate that the exposure of CALR on the surface of dying cells and the secretion of soluble ICD-associated DAMPs (ATP, HMGB1, and type I IFN) are all required for the immunogenicity of ECP-driven RCD. Thus, ECP-driven ICD exhibits large mechanistic overlaps with ICD caused by immunogenic chemotherapy, radiation therapy delivered according to specific doses and fractionation, as well as hypericin-based photodynamic therapy^14^.

## Discussion

ECP is widely employed for the management of cutaneous manifestations from CTCL^1^. Classically, such a favorable clinical activity was attributed to the existence of a therapeutic window in which ECP is cytotoxic for circulating CTCL cells (and lymphocytes), but not monocytes^7^. However, the fact that only 20–30% of circulating cells are actually exposed to ECP strongly suggested that additional mechanisms would underlie the therapeutic efficacy of ECP. Indeed, we recently demonstrated that ECP efficiently favors the conversion of circulating monocytes into physiological, cytokine-independent DCs as a consequence of physical monocyte-platelet interactions at the ECP plate surface^12^. However, the mechanisms through which ECP would provide such DCs with tumor-associated antigens (TAAs) in the context of immunostimulatory signals remained poorly defined. Here, we provide the first demonstration that ECP drives bona fide ICD. Indeed, while previous studies demonstrated that highly toxic UVC doses (100J) can trigger ICD^33^, and that CD4^+^ T cells subjected to 8-MOP plus UVA irradiation drive C-X-C motif chemokine ligand 8 (CXCL8) secretion from monocytes^34^ (knowing that CXCL8 is involved in the perception of cell death as immunogenic)^35^, the ability of ECP-treated cancer cells to initiate adaptive, tumor-specific immunity remained unexplored.

Indeed, cancer cells succumbing to 8-MOP plus 4J UVA in vitro emitted key signals involved in the perception of RCD as immunogenic, including CALR exposure on the plasma membrane as well as ATP, HMGB1, and type I IFN release (Figs. 2 and 3). Moreover, malignant cells exposed to 8-MOP plus 4J UVA irradiation were efficiently taken up by monocytes, which in turn acquired the ability to cross-prime CD8^+^ T cells against TAAs (Fig. 4). Consistent with this, cancer cells succumbing to 8-MOP plus 4J UVA light in vitro were sufficient—upon subcutaneous inoculation into syngeneic immunocompetent mice—to establish immunological protection against a subsequent challenge with living cancer cells of the same (but not different) type (Fig. 5). Finally, such a prophylactic effect was lost when the cancer cells used for vaccination were depleted of CALR or HMGB1, as well as when exposure to 8-MOP plus 4J UVA irradiation was performed in the presence of an ATP-degrading enzyme or a monoclonal antibody blocking type I IFN receptors (Fig. 6). Altogether, these findings suggest that the therapeutic effects of ECP against CTCL may reflect its ability to drive anticancer immunity. Interestingly, ECP-driven ICD resembles its radiotherapy-driven counterparts not only because it mechanistically depends on CALR exposure as well as ATP, HMGB1, and type I IFN release^14^, but also because it is compromised at high radiation doses (Supplementary Fig. 1). Indeed, radiation therapy can efficiently drive the secretion of type I IFN downstream of cyclic GMP-AMP synthase (CGAS) activation only when administered at doses that fail to upregulate the cytosolic nuclease three prime repair exonuclease 1 (TREX1)^21,36^. Thus, it will be interesting to investigate whether 8-MOP plus 4J UVA irradiation enables the accumulation of cytosolic DNA species and which are the UVA threshold doses for TREX1 upregulation.

Importantly, ECP is also used for the therapy of (auto)immune disorders, including (but not limited to) acute graft-versus-host disease (GVHD), another setting in which ECP was believed to mediate therapeutic effects upon the depletion of pathogenic lymphocytes from the peripheral blood^37^. Although DAMP emission by lymphocytes succumbing to 8-MOP plus UVA light in vitro has recently been suggested to participate in the rapid, immunologically silent clearance of these cells by monocytes^38^, we surmise that additional mechanisms may explain the clinical efficacy of ECP in GVHD patients. Indeed, data from the Glimcher group demonstrate that monocytes experiencing ER stress are impaired in their capacity to cross-present extracellular antigens to CD8^+^ T cells^39^. Moreover, 8-MOP plus UVA irradiation efficiently drive CALR exposure (Fig. 2), which depends on the activation of an ER stress response^40^. Finally, only a fraction of pathogenic lymphocytes is exposed to ECP^1^. Thus, it is tempting to hypothesize that although monocytes do not succumb to ECP, they may acquire robust tolerogenic functions that effectively quench GVHD. This possibility remains to be experimentally addressed. Irrespective of the aforementioned open questions, our data provide the first demonstration that ECP induces bona fide ICD. Besides uncovering (part of) the mechanisms whereby ECP mediates clinical activity in CTCL patients, these findings may be instrumental for the development of improved ECP protocols that may benefit patients with other circulating neoplasms.

## Supplementary information

Suppl. Fig .1

High UVA doses compromise ICD driven by 8-MOP plus UVA light

## Data Availability

The datasets used and/or analyzed during the current study are available from the corresponding author on reasonable request.
