# Supplementary figures and images for: Extracorporeal photochemotherapy induces bona fide immunogenic cell death
Source: Cell Death Dis. 2019 Aug 2;10(8):578. doi: 10.1038/s41419-019-1819-3 (PMC6675789; doi:10.1038/s41419-019-1819-3)

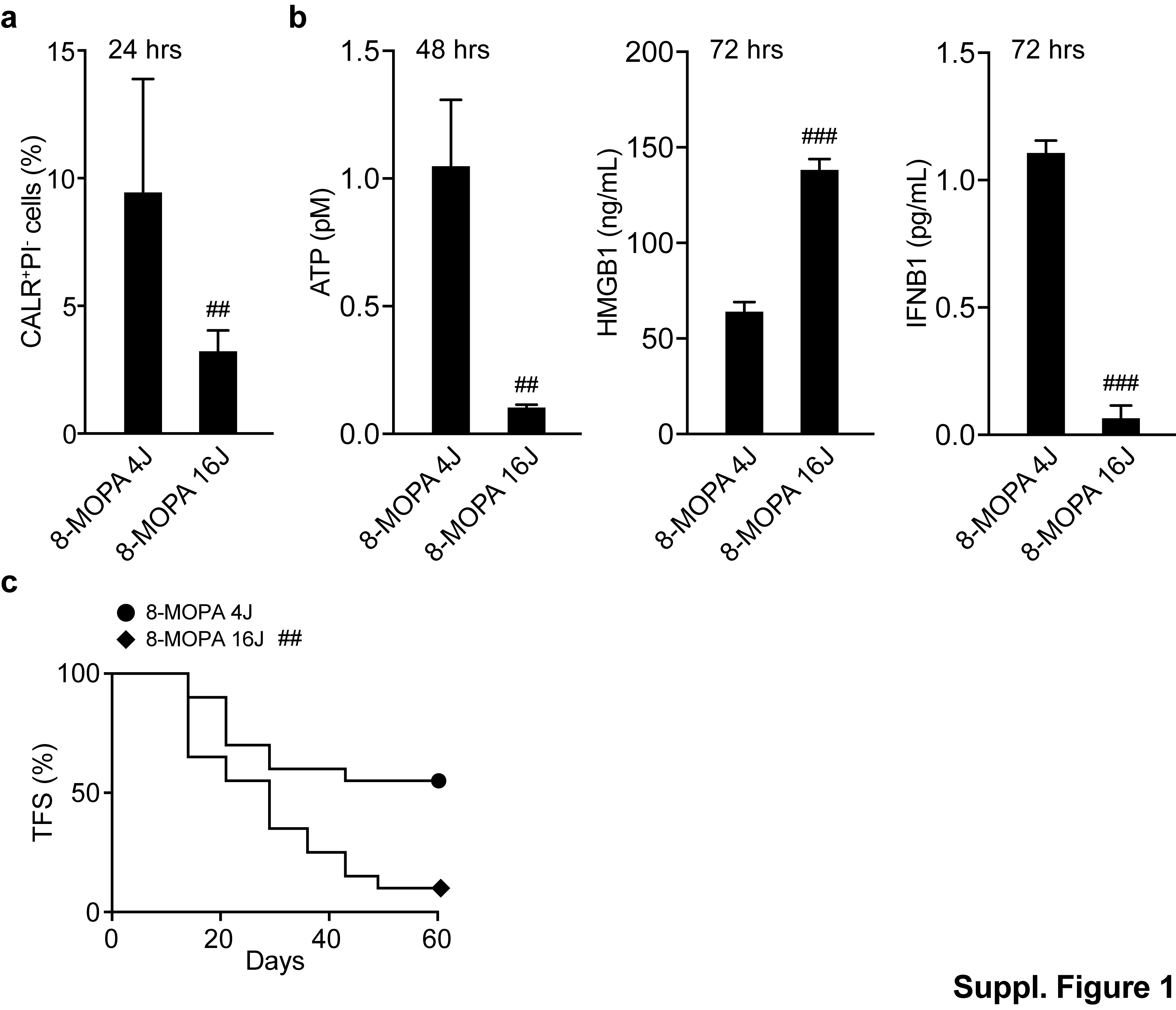

Supplement: Supplementary file 1 — Suppl. Fig .1 [file 41419_2019_1819_MOESM1_ESM.tif]
