# Supplementary material for: Extracorporeal photochemotherapy induces bona fide immunogenic cell death
Source: Cell Death Dis. 2019 Aug 2;10(8):578. doi: 10.1038/s41419-019-1819-3 (PMC6675789; doi:10.1038/s41419-019-1819-3)
Supplement: Supplementary file 2 — High UVA doses compromise ICD driven by 8-MOP plus UVA light [file 41419_2019_1819_MOESM2_ESM.docx]

***Supplemental Figure 1.* High UVA doses compromise ICD driven by 8-MOP plus UVA light.**

**A.** Percentage of CALR^+^PI­^-^ B16-OVA cells upon exposure to 8-methoxypsoralen (8-MOP) plus 4J or 16J UVA light and culture in control conditions for 24 hrs. n=6; ^##^*p*<0.01 (Student’s t test), as compared to cells exposed to 8-MOP plus 4J UVA light.

**B.** Amounts of ATP, high mobility group box 1 (HMGB1) and interferon beta 1 (IFNB1) in the supernatants of B16-OVA cells exposed to 8-MOP plus 4J or 16J UVA light and then cultured in control conditions for the indicated time. n=8; ^##^*p*<0.01, ^###^*p*<0.001 (Student’s t test), as compared to cells exposed to 8-MOP plus 4J UVA light.

**C.** Tumor-free survival (TFS) of C57BL/6 mice upon vaccination with B16-OVA cells exposed to 8-MOP plus 4J or 16J UVA light and cultured for 24 hrs in control conditions, followed (one week later) by the contralateral inoculation of living B16-OVA cells. Quantitative results are reported. n=20 mice per group; ^##^*p*<0.01 (Log-rank test), as compared to mice vaccinated with B16-OVA cells responding to 8-MOP plus 4J UVA light.
